# External Evaluation of Population Pharmacokinetic Models of Busulfan in Chinese Adult Hematopoietic Stem Cell Transplantation Recipients

**DOI:** 10.3389/fphar.2022.835037

**Published:** 2022-07-07

**Authors:** Huiping Huang, Qingxia Liu, Xiaohan Zhang, Helin Xie, Maobai Liu, Nupur Chaphekar, Xuemei Wu

**Affiliations:** ^1^ Department of Pharmacy, Fujian Medical University Union Hospital, Fuzhou, China; ^2^ School of Pharmacy, Fujian Medical University, Fuzhou, China; ^3^ College of Arts and Sciences, University of Virginia, Charlottesville, VA, United States; ^4^ Department of Pharmaceutical Sciences, School of Pharmacy, University of Pittsburgh, Pittsburgh, PA, United States

**Keywords:** busulfan, population pharmacokinetic model, external evaluation, hematopoietic stem cell transplantation, precision medicine

## Abstract

**Objective:** Busulfan (BU) is a bi-functional DNA-alkylating agent used in patients undergoing hematopoietic stem cell transplantation (HSCT). Over the last decades, several population pharmacokinetic (pop PK) models of BU have been established, but external evaluation has not been performed for almost all models. The purpose of the study was to evaluate the predictive performance of published pop PK models of intravenous BU in adults using an independent dataset from Chinese HSCT patients, and to identify the best model to guide personalized dosing.

**Methods:** The external evaluation methods included prediction-based diagnostics, simulation-based diagnostics, and Bayesian forecasting. In prediction-based diagnostics, the relative prediction error (PE%) was calculated by comparing the population predicted concentration (PRED) with the observations. Simulation-based diagnostics included the prediction- and variability-corrected visual predictive check (pvcVPC) and the normalized prediction distribution error (NPDE). Bayesian forecasting was executed by giving prior one to four observations. The factors influencing the model predictability, including the impact of structural models, were assessed.

**Results:** A total of 440 concentrations (110 patients) were obtained for analysis. Based on prediction-based diagnostics and Bayesian forecasting, preferable predictive performance was observed in the model developed by Huang et al. The median PE% was -1.44% which was closest to 0, and the maximum F_20_ of 57.27% and F_30_ of 72.73% were achieved. Bayesian forecasting demonstrated that prior concentrations remarkably improved the prediction precision and accuracy of all models, even with only one prior concentration.

**Conclusion:** This is the first study to comprehensively evaluate published pop PK models of BU. The model built by Huang et al. had satisfactory predictive performance, which can be used to guide individualized dosage adjustment of BU in Chinese patients.

## 1 Introduction

Busulfan (BU) is a bi-functional DNA-alkylating agent used in conditional regimens in patients undergoing hematopoietic stem cell transplantation (HSCT) (Lawson et al., 2020). It is usually combined with other chemotherapeutic drugs, such as cyclophosphamide, cytarabine, and fludarabine (Chen et al., 2018; Khalil et al., 2018). It can dampen the immune system response to avoid graft rejection and provide conditions favorable for the implantation of normal hematopoietic stem cells.

Both the U.S. Food and Drug Administration (FDA) and European Medicines Agency (EMA) recommend an initial intravenous BU dose of 0.8 mg/kg for adults every 6 h for 4 days. The distribution of BU in adults is very rapid with an average half-life of 0.051 h (Hassan et al., 1994). BU is conjugated with glutathione (GSH) followed by intramolecular rearrangement to the GSH analog γ–glutamyl-dehydroalanyl-glycine (EdAG), which is mainly catalyzed by the enzyme glutathione S-transferase (GSTs) in the liver (Gibbs et al., 1996; Scian and Atkins, 2015). A high inter-individual variability is observed in the elimination half-life, varying from 0.97 to 7.2 h (Grochow et al., 1989). The excretion of unchanged drug into the urine is less (about 1–2%) (Ehrsson et al., 1983; Hassan et al., 1989).

BU has a narrow therapeutic window. Fifty percent inter- and intra-individual variability in pharmacokinetics (PK) has been reported in the literature (Hassan, 1999; Veal et al., 2012; Paci et al., 2014; Marsit et al., 2020). Studies have shown that area under the concentration-time curve (AUC_ss_) or plasma concentration (C_ss_) at steady-state is closely associated with the efficacy and toxicity (Bartelink et al., 2009; Ansari et al., 2014; Bartelink et al., 2016; Feng et al., 2020; Hill et al., 2020). Based on the plasma pharmacokinetics of BU, therapeutic drug monitoring (TDM) is recommended to improve engraftment (Kanda, 2018; Takachi et al., 2019). There is no recommended therapeutic window for BU in China now. FDA suggests that AUC of BU should be between 900–1,350 ± 5% μmol/L × min, while a therapeutic range of 900–1,500 μmol/L × min is recommended by EMA with every 6-h dosing (Nguyen et al., 2004; Palmer et al., 2016). The Practice Guidelines Committee of the American Society of Blood or Marrow Transplantation (ASBMT) also highlights the necessity for BU TDM (Bubalo et al., 2014; Tesfaye et al., 2014). It emphasizes that personalized BU dosing needs to be considered to minimize sinusoidal obstruction syndrome, lower graft rejection, and relapse rates (Bubalo et al., 2014). TDM guided BU dosing is routinely conducted in some institutions (Philippe et al., 2016; Shukla et al., 2020).

There are usually two ways to adjust dosing. First is the conventional PK-guided dose adjustment routinely performed in the clinical practice. AUC or Css can be calculated either by multiple pharmacokinetic samples (at least five samples) or a reliable limited sampling strategy (LSS) (Malär et al., 2011; Davis et al., 2019). Dosage can be adjusted by comparing the current AUC or Css with the target values. LSS has the advantage of predicting AUC with 2-4 samples (Huang et al., 2017; Teitelbaum et al., 2020). Recently, personalized dosing strategy based on population pharmacokinetic (pop PK) model coupled with Bayesian forecasting has become popular (Chaivichacharn et al., 2020; Gil Candel et al., 2020). It can obtain individual PK parameters with 1-2 concentrations per patient to get the individualized dosing via maximum a posteriori (Thomson and Whiting, 1992). It can overcome the inconvenience of multiple sampling. Many computer programs with built-in pop PK models have emerged (Felton et al., 2014; Ramos-Martin et al., 2017; Frymoyer et al., 2020; Kantasiripitak et al., 2020). These kind of computer-assisted decision tools usually have a user-friendly interface for application by physicians or pharmacologist.

Over the last decades, several pop PK models of BU have been developed (Nguyen et al., 2006; Salinger et al., 2010; Choe et al., 2012; Choi et al., 2015; Wang et al., 2015; Su et al., 2016; Wu et al., 2017; Huang et al., 2019; Sun et al., 2020), while differences exist between the various models. When these pop PK models are applied to guide individualized dosing in Chinese or other ethnic populations, the accuracy of their prediction needs to be explored and then only the fully validated model can be used to guide drug dosing. This study aimed to evaluate the predictive performance of the published intravenous BU pop PK models in adults. In order to identify which model is the best choice to guide personalized dosing in Chinese HSCT patients, the external predictability of the models was assessed using data obtained from Chinese adult patients undergoing HSCT in our center.

## 2 Methods

### 2.1 Review of the Published Pop PK Studies

An extensive literature search was performed using PubMed, China National Knowledge Infrastructure (www.cnki.net), and Wanfang Data (www.wanfangdata.com.cn) for studies up to 31 October 2020, using the keywords “Busulfan” and “Population Pharmacokinetics”. Studies were included if they contained a pop PK model of intravenous BU in adults and were written in Chinese or English. The reference lists of the selected literatures should also be checked for additional studies. If the essential parameters of the pop PK models (typical value of CL, inter-individual variability of CL, etc.) were missing, the studies were excluded. On the occasions where studies were developed with overlapping data or cohorts, only the one with the largest study cohort was included. Published pop PK models were re-coded and the parameters were obtained from the final model in the literature.

### 2.2 Software

The external evaluation was conducted with non-linear mixed-effects modelling software package NONMEM version 7.5 (ICON Development Solutions, MD, United States), using Pirana 2.9.7 and Perl-speaks-NONMEM (PsN) Toolkit 4.8.1 as the modelling interface. Data handling, visualization and statistics were performed in R 4.0.0 (R Foundation for Statistical Computing, Vienna, Austria) and RStudio 1.2.5001 (RStudio Inc. Boston, MA, United States).

### 2.3 Study Cohort of External Evaluation

#### 2.3.1 External Dataset

The concentrations of 110 adult patients who received BU intravenously prior to HSCT at Fujian Medical University Union Hospital from March 2013 to May 2018 were collected as the external data. The study protocols were approved by the Ethics Committee of the Fujian Medical University Union Hospital and written informed consent was obtained from all the subjects. The demographic characteristics, biochemistry data, genetic polymorphisms information, and concomitant drugs are summarized in Table 1.

**TABLE 1:** Demographics of external dataset.

	Mean ± SD/N (%)	Range
Demographic		
WT (kg)	62.6 ± 12.1	42.0–100
AIBW (kg)	59.0 ± 7.93	42.0–75.3
IBW (kg)	60.8 ± 5.94	47.5–73.7
BSA (m2)	1.69 ± 0.186	1.31–2.19
BMI (kg/m^2^)	22.6 ± 3.74	14.04–33.80
Age (years)	34.8 ± 11.9	19–65
Gender (M/F)	60/50	—
Height (cm)	166.1 ± 8.12	147–183
Biochemistry		
Serum creatinine (μmol/L)	58.9 ± 16.8	25.0–117
Creatinine clearance (mL/min)	134 ± 36.1	62.9–245
Aspartate transaminase (IU/L)	27.7 ± 15.9	6–100
Alkaline phosphatase (IU/L)	62.0 ± 18.3	28.0–129
Gamma glutamyl transferase (IU/L)	38.2 ± 39.2	8.0–288
Serum albumin (g/L)	38.8 ± 5.19	28.0–49.7
Lactic dehydrogenase (IU/L)	229 ± 104	90.0–607
Total bilirubin (μmol/L)	13.27 ± 7.90	1.90–57.3
Alanine transaminase (IU/L)	38.3 ± 34.7	6.0–165
Genetic polymorphisms in GSTA_1_ ^a^ ^,^ ^b^		
GG (^a^A^a^A)	58 (52.7%)	—
GA (^a^A^a^B)	14 (12.7%)	—
AA (^a^B^a^B)	0 (0%)	—
Diagnosis^b^		
Acute myeloid leukemia (AML)	44 (40.0%)	—
Acute lymphoblastic leukemia (ALL)	34 (30.9%)	—
Chronic myeloid leukemia (CML)	10 (0.09%)	—
Myelodysplastic syndrome (MDS)	10 (0.09%)	—
Miscellaneous	12 (10.9%)	—
Concomitant drug^b^		
Tropisetron	13 (11.8%)	—
Palonosetron	99 (90.0%)	—
Phenytoin (PHT)	98 (89.1%)	—
Micafungin sodium	19 (17.3%)	—
Voriconazole (VOR)	20 (18.2%)	—
Cyclosporine A (CSA)	67 (60.9%)	—

a Thirty-eight patients in external dataset have no genetic polymorphisms information.

b The category variables such as genetic polymorphisms in GSTA_1_, diagnosis, and concomitant drug are presented as n (%).

#### 2.3.2 Dosing Regimen and Sampling

All patients received 0.8 mg/kg of BU every 6 h for 4 or 3 days, combined with other chemotherapeutic drugs (Cyclophosphamide, Fludarabine, etc.) as the conditional regimens prior to HSCT. Oral phenytoin (5–10 mg/kg/d) was given to prevent seizures. Cyclosporin was administered intravenously before transplantation. Anti-emetics and antifungal drugs were used during chemotherapy, depending on the actual clinical situation.

The dosage of BU with an infusion over 2 h was determined based on the adjusted ideal body weight (AIBW) which was calculated using the following formulas: IBW = height^2^ × 22/10,000 and AIBW = IBW+0.25 × (ABW-IBW). If actual body weight (ABW) ≤ IBW, ABW would be equal to IBW (Wu et al., 2017). Intensive blood samples were collected from 28 patients at 0.5, 1, 2, 3, 4, 5 and 6 h after the start of the first dose infusion as well as pre-infusion of the fifth dose and 2 h after the start of the fifth dose infusion. Considering the convenience of clinical practice, a limited sampling strategy was conducted in the other 82 patients at 1, 3, and 5 h after the start of the first dose. A total of 440 concentrations were obtained for analysis. The plasma concentrations of BU were determined using high-performance liquid chromatography-mass spectrometry (HPLC-MS/MS). The calibration standards were linear over concentrations ranging from 0.05 to 2.5 μg/ml. The lower limit of detection was 3 ng/ml at which the signal level of BU reached at least 3 times the signal noise of the baseline. The single nucleotide polymorphism (SNPs) of GSTs were determined by matrix-assisted laser desorption/ionization-time of flight (MALDI-TOF-MS).

### 2.4 External Evaluation

#### 2.4.1 Prediction-Based Diagnostics

The relative prediction error (PE%) was calculated by comparing the population predicted concentration (PRED) with the observations (OBS) using the Eq. A. If PEs% departed from the normal distribution, the median prediction error (MDPE, median PE%) was calculated to reflect accuracy. Meanwhile, the median absolute prediction error (MAPE, median |PE|%) was used to indicate precision. The percentage of PE% falling within the ±20% and ± 30% (F_20_, F_30_) were computed to represent combination index of accuracy and precision (Mao et al., 2018). The candidate model was considered to be clinically acceptable when the standards of MDPE≤±15%, MAPE≤30%, F_20_ > 35% and F_30_ > 50% were reached.

$$\mathrm{PE\%}=\left(\frac{\mathrm{PRED-OBS}}{\mathrm{OBS}}\right)\mathrm{\times 100 Equation}\left(A\right)$$ (1)

#### 2.4.2 Simulation-Based Diagnostics

The prediction- and variability-corrected visual predictive check (pvcVPC) and the normalized prediction distribution error (NPDE) were executed for the simulation-based diagnostics. The pvcVPCs were simulated by the PsN toolkit. The dataset was simulated for 2000 times (Zhao et al., 2016). The 95% confidence intervals (CI) for the median and the 5th and 95th percentiles of the simulations were calculated and compared with the prediction- and variability-corrected observations. NPDE contains four statistical tests (Wilcoxon signed rank test, Fisher test, Shapiro-Wilks test, and Global test) to verify whether NPDE follows a standard normal distribution N (0,1). The results of NPDE were output by R software statistically and graphically.

#### 2.4.3 Bayesian Forecasting

The Maximum a Posterior Bayesian (MAPB) forecasting was used to evaluate the effect of previous observations on model predictability (Zhang et al., 2019). A total of 107 patients with ≥3 observations were included in the evaluation. The individual predictions (IPRED) of observation for all patients were predicted by giving one to four prior observations, respectively. The individual PE% (IPE%) was computed by the following Eq. B. Similar to prediction-based diagnostics, median IPE% (MDIPE), median absolute IPE% (MAIPE), F_20_ and F_30_ of IPE% (IF_20_, IF_30_) were computed to reflect the overall prediction performance of the model.

$$\mathrm{IPE\%}=\left(\frac{\mathrm{IPRED-OBS}}{\mathrm{OBS}}\right)\mathrm{\times 100 Equation}\left(B\right)$$ (2)

### 2.5 Impact of Structural Model and Covariates

All structural models of the published studies had been generalized owing to the considerable effect on the prediction. Their impacts on the predictive performance were evaluated with or without major significant covariates. The covariates were screened using a stepwise method, which is consistent with the published pop PK studies. Three evaluation methods, the predication- and simulation-based diagnostics, and Bayesian forecasting, were applied.

## 3 Results

### 3.1 Reviews of the Published Pop PK Analyses

A total of nine BU pop PK studies were published (Nguyen et al., 2006; Takama et al., 2006; Salinger et al., 2010; Choe et al., 2012; Choi et al., 2015; Wang et al., 2015; Su et al., 2016; Huang et al., 2019; Sun et al., 2020). Two studies were excluded. One was due to missing key parameters, another one involved inter-occasion variability but the sampling times were in the ninth and 13th dose, which is different from the external dataset (Nguyen et al., 2006; Takama et al., 2006). Seven studies were eventually retained for evaluation (Salinger et al., 2010; Choe et al., 2012; Choi et al., 2015; Wang et al., 2015; Su et al., 2016; Huang et al., 2019; Sun et al., 2020) and the details are listed in Table 2. Three of them were performed in China (Su et al., 2016; Huang et al., 2019; Sun et al., 2020), two in Korea (Choe et al., 2012; Choi et al., 2015), and two in the United States (Salinger et al., 2010; Wang et al., 2015). Only one study was a multicenter study (Wang et al., 2015). Six pop PK models were fitted with one compartmental model (1-CMT) (Salinger et al., 2010; Choe et al., 2012; Choi et al., 2015; Wang et al., 2015; Huang et al., 2019; Sun et al., 2020), and only one was fitted with two compartmental model (2-CMT) (Su et al., 2016). The covariates involved in the published final CL models were body weight (BW), body surface area (BSA), serum creatinine (Scr), GSTA_1_ genotype, and gender (SEX). BW, as the most recognized covariate, was incorporated in three pop PK models (Choe et al., 2012; Choi et al., 2015; Su et al., 2016). There were two pop PK studies that found no covariates impacting CL (Salinger et al., 2010; Huang et al., 2019). In two studies, the relationship between GSTA_1_ genotype and CL was taken into consideration, and GSTA_1_ genotype was incorporated into their model (Choi et al., 2015; Sun et al., 2020). The typical CL and V of a 60–65 kg male varied from 7.3–14.2 L/h and 30.9–64.1 L, respectively. This discrepancy across the seven studies needs further investigations.

**TABLE 2:** Summary of published population pharmacokinetic studies of busulfan in adult hematopoietic stem cell transplantation recipients.

Study (publication year)	Country (single/Multiple sites)	Number of Patients (Male/Female)	Sampling schedule (number of samples)	Bioassay	Structural model	PK Parameters and formula	BSV(%)	Residual error
Choi et al. (2015)	Korean (Single)	36 (21/15)	IS(101)	HPLC/MS/MS	1-CMT	CL = 11.0 × (BW/60)^0.843^×e^(−0.161) ×GSTA1^ (L/h)	14.7	15.3%
Choi et al. (2015)						V_d_ = 42.4 (L)	25.6	
Wang et al. (2015)	The U.S. (Multiple)	207(NA)	IS(2,454)	NA	1-CMT	CL = 7.74 × (BSA −2.0) + 12.7 (L/h)	13.7	8.65%
Wang et al. (2015)						V_d_ = 32.8 × (BSA −2.0) + 50.3 for male (L)	9.49	
						V_d_ = 32.8 × (BSA −2.0) + 46.3 for female (L)	—	
Choe et al. (2012)	Korean (Single)	60 (37/23)	IS(295)	LC/MS/MS	1-CMT	CL = 0.947 × ABW^0.5^ (L/h)	16	6.3%
Choe et al. (2012)						V_d_ = 3.610 × ABW^0.5^×(1 + SEX × 0.105) (L)	9	
Salinger et al. (2010) (Salinger et al., 2010)	The U.S. (Single)	37 (21/16)	IS(777)	GC/MS	1-CMT	CL = 0.179 (L/h/kg)	19.7	8.6%
						V_d_ = 0.723 (L/kg)	15.6	14.06 ng/ml
Huang et al. (2019) (Huang et al., 2019)	China (Single)	20 (11/9)	IS(280)	LC/MS/MS	1-CMT	CL = 12.02 (L/h)	15	20.3%
						V_d_ = 50.94 (L)	19	
Sun et al. (2020)	China (Single)	43 (32/11)	IS(488)	LC/MS/MS	1-CMT	CL = 14.2 × (1 + (-0.214)× GSTA1 (L/h)	14.6	-14.1%
Sun et al. (2020)						V_d_ = 64.1 (L)	16.7	
Su et al. (2016)	China (Single)	35 (23/12)	IS + LS(NA)	HPLC	2-CMT	CL = 8.11 × (WT/50)^0.726^ × 1.39^SEX^ (L/h)	18.9	136.01 ng/ml
Su et al. (2016)						V_1_ = 24.9×(CRE/53)^0.507^×(WT/50)^1.35^ (L)	31.1	
						Q = 22.2 (L/h)	69.2	
						V_2_ = 28.1 (L)	43.3	

CRE, creatinine; WT/BW, body weight; BSA, body surface area; CL, clearance; ABW, actual body weight; 1-CMT, one-compartment model; 2-CMT, two-compartment model; HPLC, high performance liquid chromatography; LC/MS/MS, liquid chromatography tandem-mass spectrometry; GC, gas chromatography with mass selective detection; IS, intensive sampling; SS, sparse sampling; LS, limited sampling; BSV, between-subject variability; NA, not available.

### 3.2 External Evaluation

#### 3.2.1 Prediction-Based Diagnostics

The prediction-based diagnostic results were shown in Table 3. Five of seven models met all the criteria (MDPE ≤ ±20%, MAPE ≤30%, F_20_ ≥ 35%, and F_30_ ≥ 50%) (Salinger et al., 2010; Choi et al., 2015; Su et al., 2016; Huang et al., 2019; Sun et al., 2020). Taking both accuracy and precision into account, the model developed by Huang et al. (2019) showed preferable predictive performances compared to the others. The model yielded a MDPE of -1.44%, which was the closest to 0. The maximum F_20_ (57.27%) and F_30_ (72.73%) were also achieved.

**TABLE 3:** Results of prediction-based diagnostics.

Models	PE_min_ (%)	PE_max_ (%)	MDPE (%)	MAPE (%)	F_20_ (%)	F_30_ (%)
Published studies						
Choi et al. (2015)	−79.51	508.58	10.36	19.33	51.82	66.59
Wang et al. (2015)	−77.98	609.49	25.59	27.23	36.82	54.77
Choe et al. (2012)	−71.71	766.42	63.57	63.90	11.59	18.18
Salinger et al. (2010)	−64.59	943.19	−10.36	25.04	41.36	57.05
Huang et al. (2019)	−83.12	538.23	−1.44	16.25	57.27	72.73
Sun et al. (2020)	−86.24	409.11	−19.25	24.20	42.27	61.36
Su et al. (2016)	−80.37	480.88	−10.36	22.97	42.73	66.14
Impact of model structure						
1-CMT (Base Model)	−82.75	557.73	2.16	16.93	55.45	70.91
1-CMT+BSA	−82.68	511.25	0.78	15.74	57.73	71.59
2-CMT (Base Model)	−81.52	573.23	7.35	19.31	50.68	68.41
2-CMT+BSA	−81.41	519.43	5.47	17.54	55.68	70.45

PE_min_, the minimal of prediction error; PE_max_, the maximum of prediction error; MDPE (%), median prediction error; MDAE (%), median absolute prediction error; F_20_ (%) and F_30_ (%) the percentage of prediction error ≤ ±20% and ± 30%, respectively; 1-CMT, one-compartment model; 2-CMT, two-compartment model; BSA, body surface area.

#### 3.2.2 Simulation-Based Diagnostics

**FIGURE 1:**
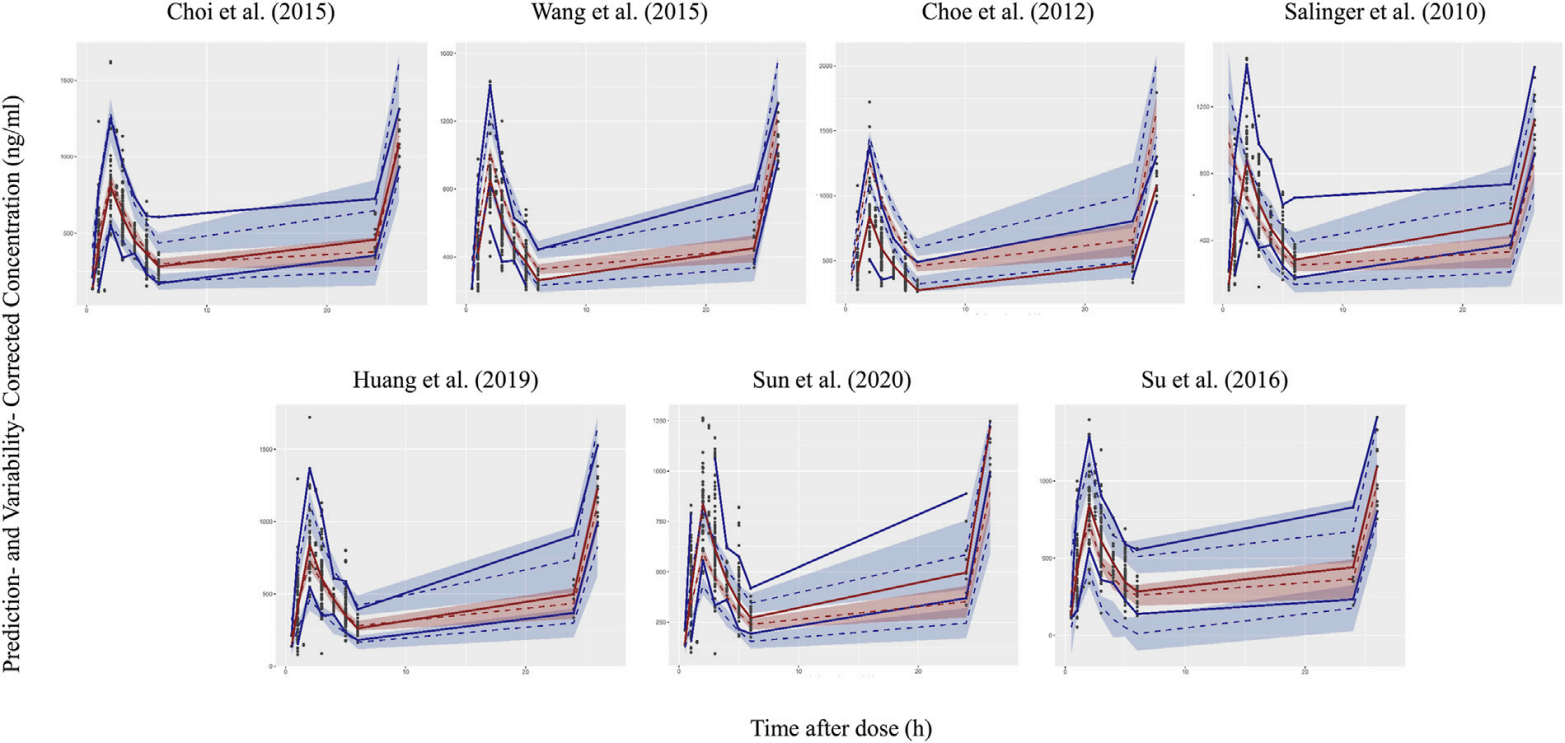
Prediction- and variability-corrected visual predictive check (pvcVPC) plots of seven published population pharmacokinetic models (Salinger et al., 2010; Choe et al., 2012; Choi et al., 2015; Wang et al., 2015; Su et al., 2016; Huang et al., 2019; Sun et al., 2020). The middle dashed line represents the median prediction- and variability-corrected predictions. The middle semitransparent field represents a simulation-based 95% confidence interval (CI) for the median. Upper and lower dash lines represent the corrected observed 95th and fifth percentiles and semitransparent fields represent a simulation-based 95% CI for the corresponding model predicted percentiles. The solid lines represent the median, 95th and 5th percentiles of observations.

**FIGURE 2:**
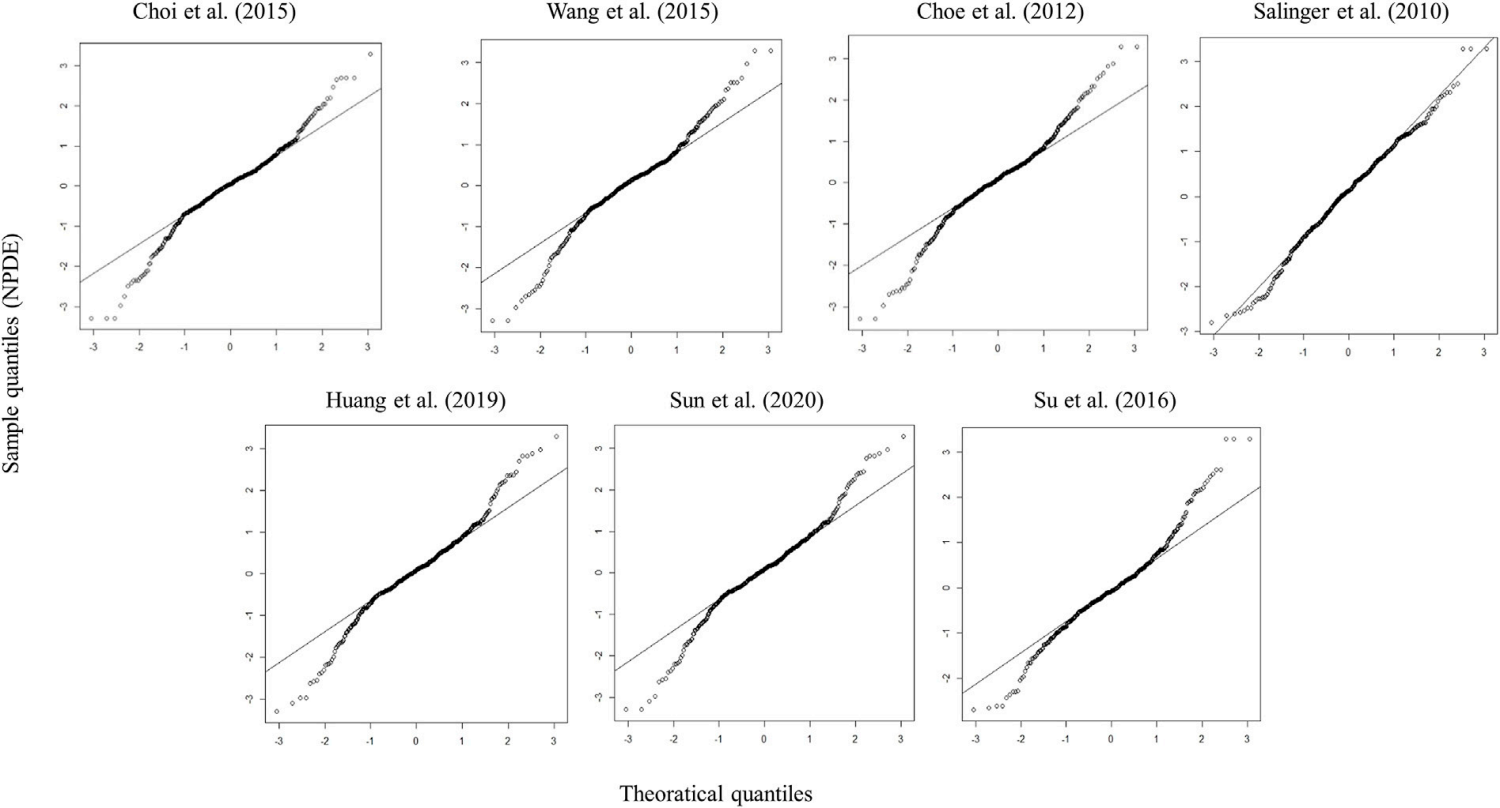
Quantile–quantile plots (the distribution of the NPDE against theoretical distribution) of seven published population pharmacokinetic models (Salinger et al., 2010; Choe et al., 2012; Choi et al., 2015; Wang et al., 2015; Su et al., 2016; Huang et al., 2019; Sun et al., 2020).

Four models showed an un-ignorable difference between the observations and simulations in pvcVPC (Salinger et al., 2010; Choe et al., 2012; Wang et al., 2015; Sun et al., 2020). The model developed by Choi et al. (Choi et al., 2015) and Huang et al. (Huang et al., 2019) performed better than the other models in pvcVPC (Figure 1). Regarding the standard normal distribution of NPDE, NPDE plot of the model by Salinger et al. (Salinger et al., 2010) seemed to be better than other models as shown in Figure 2. However, Supplementary Table S2 presented the results of four statistical tests, model built by Choi et al. passed Wilcoxon signed rank test and Fisher test, the other models only passed one statistical test (*p* ≥ 0.05). No model satisfied all statistical test, which means all models failed in NPDE diagnostics.

#### 3.2.3 Bayesian Forecasting

**FIGURE 3:**
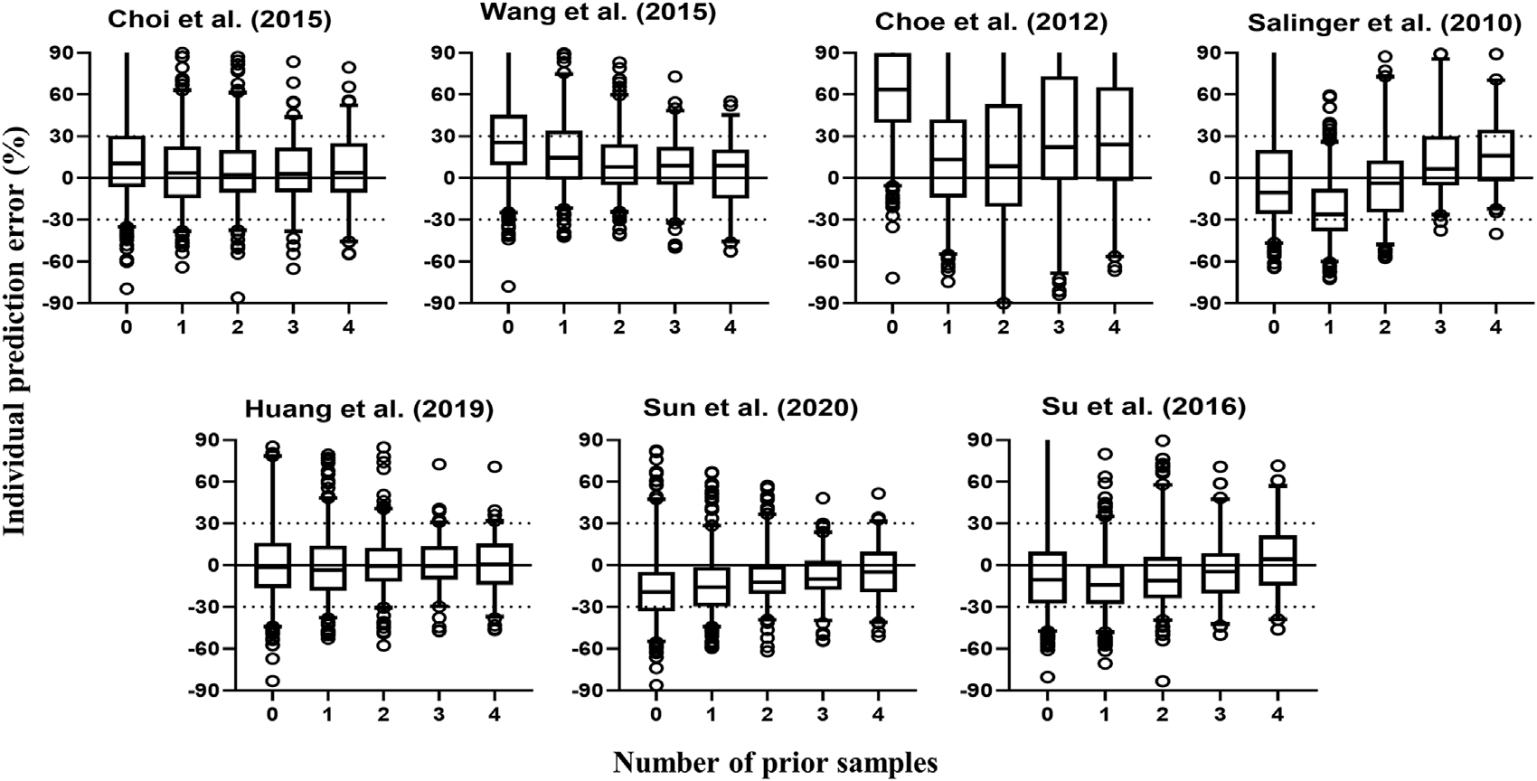
Box plots of individual relative prediction error (IPE%) with Bayesian forecasting for seven published population pharmacokinetic models (Salinger et al., 2010; Choe et al., 2012; Choi et al., 2015; Wang et al., 2015; Su et al., 2016; Huang et al., 2019; Sun et al., 2020) in different scenarios (0 represents prediction without prior information and 1-4 represents with prior one to four observations, respectively). In scenario n, prior n observations were used to estimate the individual prediction and it was then compared with the corresponding observation.

Figure 3 contains box plots of IPE% with Bayesian forecasting for seven published pop PK models in different scenarios (Salinger et al., 2010; Choe et al., 2012; Choi et al., 2015; Wang et al., 2015; Su et al., 2016; Huang et al., 2019; Sun et al., 2020). The results demonstrated that prior concentrations, even one prior concentration, improved the prediction precision and accuracy of all models, which was exhibited by the narrower range of IPEs, as well as the median of IPEs being closer to 0. Two or three prior concentrations could achieve better results. The IPRED of the model by Huang et al. (2019) demonstrated the most accurate result. With two prior concentrations, the IF_20_ and IF_30_ were 69 and 85%, respectively.

### 3.3 The Impact of Structural Models and Covariates

**FIGURE 4:**
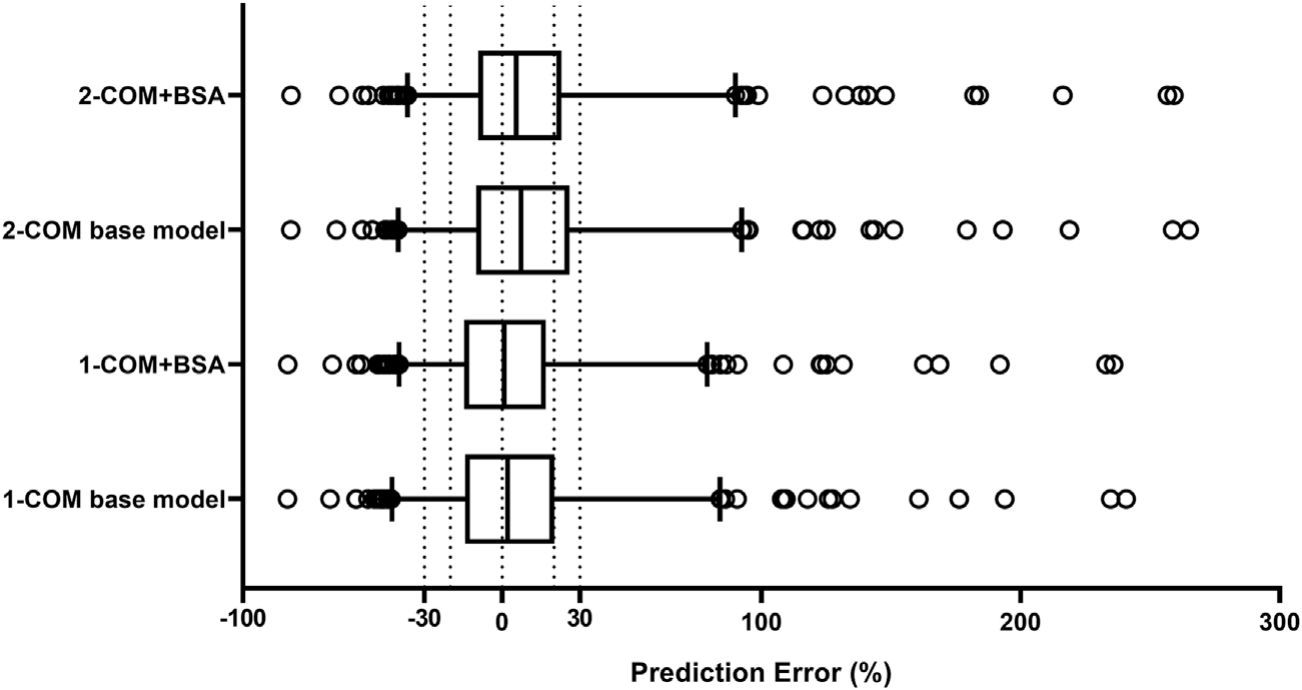
Box plots of relative prediction error (PE%) for two structural models with or without covariates. Black solid line and dotted lines are reference lines indicating PE% of 0%, ±20% and ±30%, respectively. 1-COM, one compartmental model; 2-COM, two compartmental model; BSA, body surface area.

**FIGURE 5:**
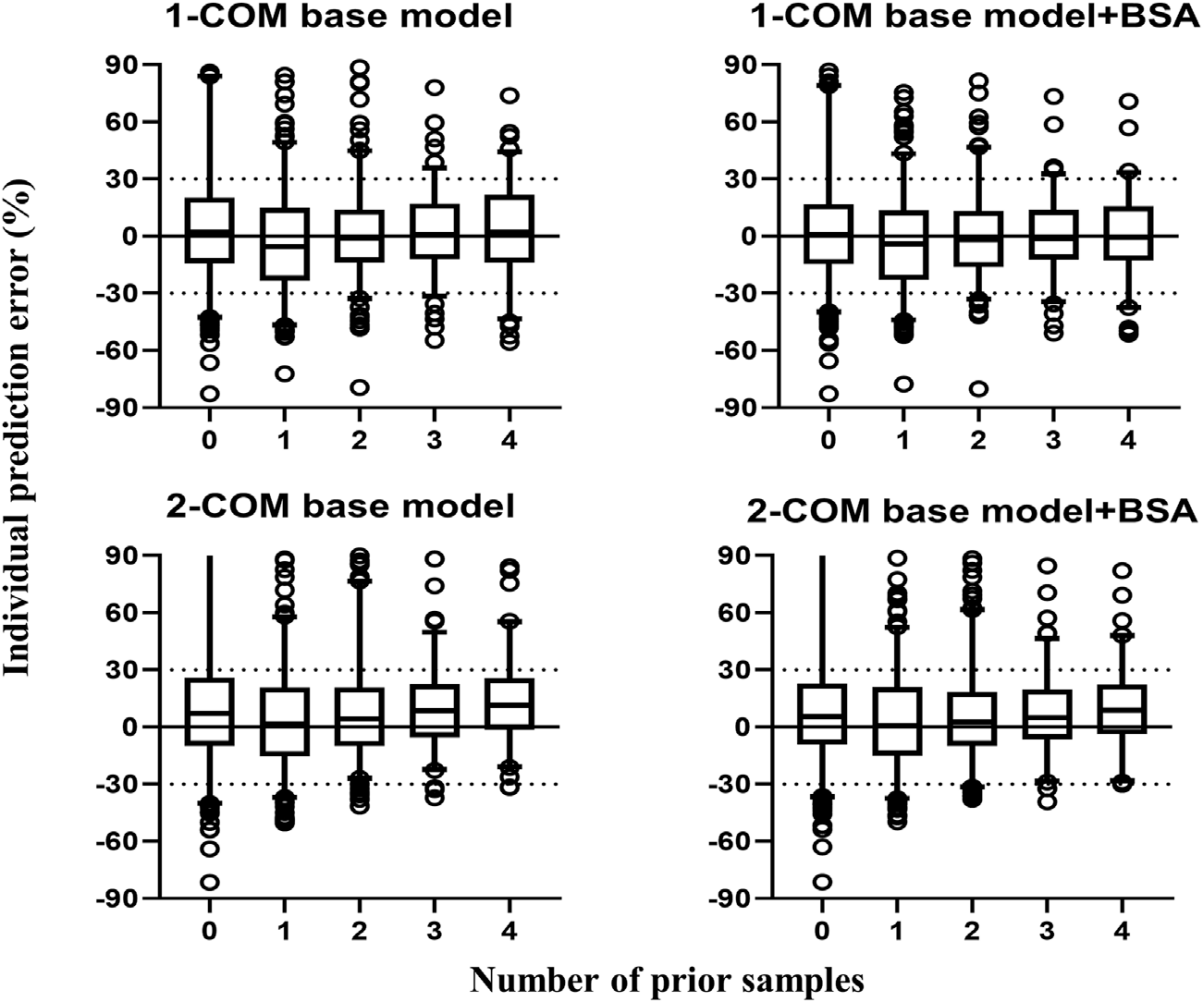
Box plots of individual relative prediction error (IPE%) with Bayesian forecasting for two structural models with or without covariates in different scenarios (0 represents prediction without prior information and 1-4 represents with prior one to four observations, respectively). In scenario n, prior n observations were used to estimate the individual prediction and it was then compared with the corresponding observation. 1-COM, one compartmental model; 2-COM, two compartmental model; BSA, body surface area.

The structural models published included the 1-CMT and 2-CMT models. The above two covariate-free structural models were first developed and evaluated. The 1-CMT model fits well with the external dataset due to a small OFV value and low variability of the PK parameters. Covariates involved in the published pop PK studies (BSA, BW, Scr, GSTA_1_ genotype, and SEX) were screened using a stepwise method. BSA was successfully included in the model based on a *p*-value of less than 0.05. Incorporation of other covariates in the model showed no significant amelioration. The results are summarized in Figure 4, 5.

## 4 Discussion

In our study, the external predictability of seven published intravenous BU pop PK models in adults (Salinger et al., 2010; Choe et al., 2012; Choi et al., 2015; Wang et al., 2015; Su et al., 2016; Huang et al., 2019; Sun et al., 2020) was explored using an independent dataset, which contained 110 patients with 440 observations. To the best of our knowledge, no similar research on BU has been published yet. All model has consistent performance in the statistical tests of NPDE. Overall, based on the results of prediction-based and Bayesian forecasting, the model built by Huang et al. (Huang et al., 2019) has satisfied predictive performance, which can be used to guide individualized dosing of BU in our center. Bayesian forecasting suggested that predictive accuracy would be improved by giving one or two prior concentrations, which indicated that the qualified published models can potentially guide personalized dosing in Chinese population.

With the progress of dose individualization, decision-making systems have been developed rapidly in recent years (Mould et al., 2016), with the characteristics of clinical compliance and predictive accuracy. The decision-making system can be designed by different forms, such as computer programs, web platforms, and applications (APPs) (Barrett et al., 2008; Hope et al., 2013; Shukla et al., 2020). They are easy to use and incorporate over 20 drugs, covering populations ranging from neonate to adult. For BU individualized dose adjustment, the computer programs BestDose (http://www.lapk.org/bestdose.php), DoseMe (https://doseme-rx.com/), and the web platform InsightRX (https://www.insight-rx.com/), NextDose (https://www.nextdose.org/) are available. All of them estimate parameters by Bayesian algorithm, but using different pop PK models. Before applying the pop PK model for BU in our center, it is necessary to externally validate the model based on institutional data.

BestDose choose a non-parametric population model of BU for individual patient therapeutic drug dose management. The model was developed in pediatric patients, using the Non-Parametric Adaptive Grid algorithm in the Pmetrics package for R. Therefore, we didn’t include it in our study. A population pharmacokinetic model by (Long-Boyle et al., 2015) for children ≥12 kg was implemented in InsightRX and available. NextDose’s recommendations use the model by (McCune et al., 2014). The above two models were built in patients ranged from infants to adults. The age range of our dataset was 19–65 years old. It may be more appropriated to evaluated them using pediatric patients. DoseMeRx supports a couple of drug models for BU. For adult, model built by (Salinger et al., 2010) is applied, which has been external evaluated in our study. However, it didn’t show satisfactory predictive performance. This maybe explain by the differences between the model development dataset (White population) and the external evaluation dataset (Chinese population). Generally speaking, the models developed in a similar population might have a superior predictive performance with external dataset because of similar ethnic background with parallel genotypes, prescribing and dietary habits. This helps to explain the superiority of the model built by Huang et al., which was developed in Chinese population.

Three diagnostics are usually used to evaluate the predictive performance of the published pop PK models. Prediction-based diagnostics is a useful method to assess the correlation of observations and simulations. The criteria are typically set as MDPE ≤ ±20%, MAPE ≤30%, F_20_ ≥ 35% and F_30_ ≥ 50% in the literature (Deng et al., 2013; Zhao et al., 2016). Simulation-based diagnostics include pvcVPC and NPDE. Compared with traditional VPCs, pvcVPC is readily applicable to data from studies with a prior and a posteriori dose adaptation (Bergstrand et al., 2011). Both pvcVPC and NPDE could allow us to correctly detect a misspecification of the model. Bayesian forecasting is usually used to adjust dosage in clinical practice with prior observations (Bhattacharjee, 2014).

With these diagnostics, the pop PK models showed different predictive performance in Chinese HSCT patients. Several factors, such as the incorporated covariates, the incorporated ways of covariates, and the characteristics of participants, may impact the predictive ability of pop PK models. In the published pop PK studies of BU, the most recognized covariate impacting CL was body size. BW/IBW/adjusted ideal body weight (AIBW)/body surface area (BSA)/body mass index (BMI) can be classified as body size (Wang et al., 2015), but just one of them can be incorporated in the formula theoretically due to collinearity. Trame et al. suggested allometric BW model and BSA model as a preferred choice for BU dosing in children, which is consistent with the study of Anderson and Holford (Anderson and Holford, 2008; Trame et al., 2011). Commonly used dosing regimen maybe based on BSA because it is most frequently used by clinicians and pharmacists in pediatric oncology (Trame et al., 2011). With regards to the structural model, we found that adding BSA to the base model significantly improved the predictive ability, the final model was CL = 11.7 × (BSA/1.69)^1.05^. This is consistent with the previous findings (Choe et al., 2012; Choi et al., 2015; Su et al., 2016). BU is mainly catalyzed by GSTs and GSTA_1_ is the main GST isoenzyme. Most of studies focused on the relationship between GSTA_1_ gene polymorphism and PK of BU, patients with the GSTA_1_ *A/*B genotype had an 8–27% lower CL than GSTA_1_ *A/*A group (Ansari et al., 2013; Yin et al., 2015; Ansari et al., 2016). However, some studies showed no association between BU exposure and GSTA_1_ genotype (Zwaveling et al., 2008; Ansari et al., 2010; Yin et al., 2015), the results remain debatable. Therefore, pharmacogenomics-based dosing of BU was not recommended by the Practice Guidelines Committee of ASBMT (Bubalo et al., 2014). It should be noted that gender was incorporated as a covariate of CL in one pop PK study and as a covariate of volume of distribution (V_d_) in two pop PK studies. According to Ansari et al. (2016), the relationship between GSTA_1_ and first BU dose PK depended on sex and Pesaro risk classification. This result may be explained by the difference in cytosolic GST activity between females and males (Miyagi et al., 2009).

**FIGURE 6:**
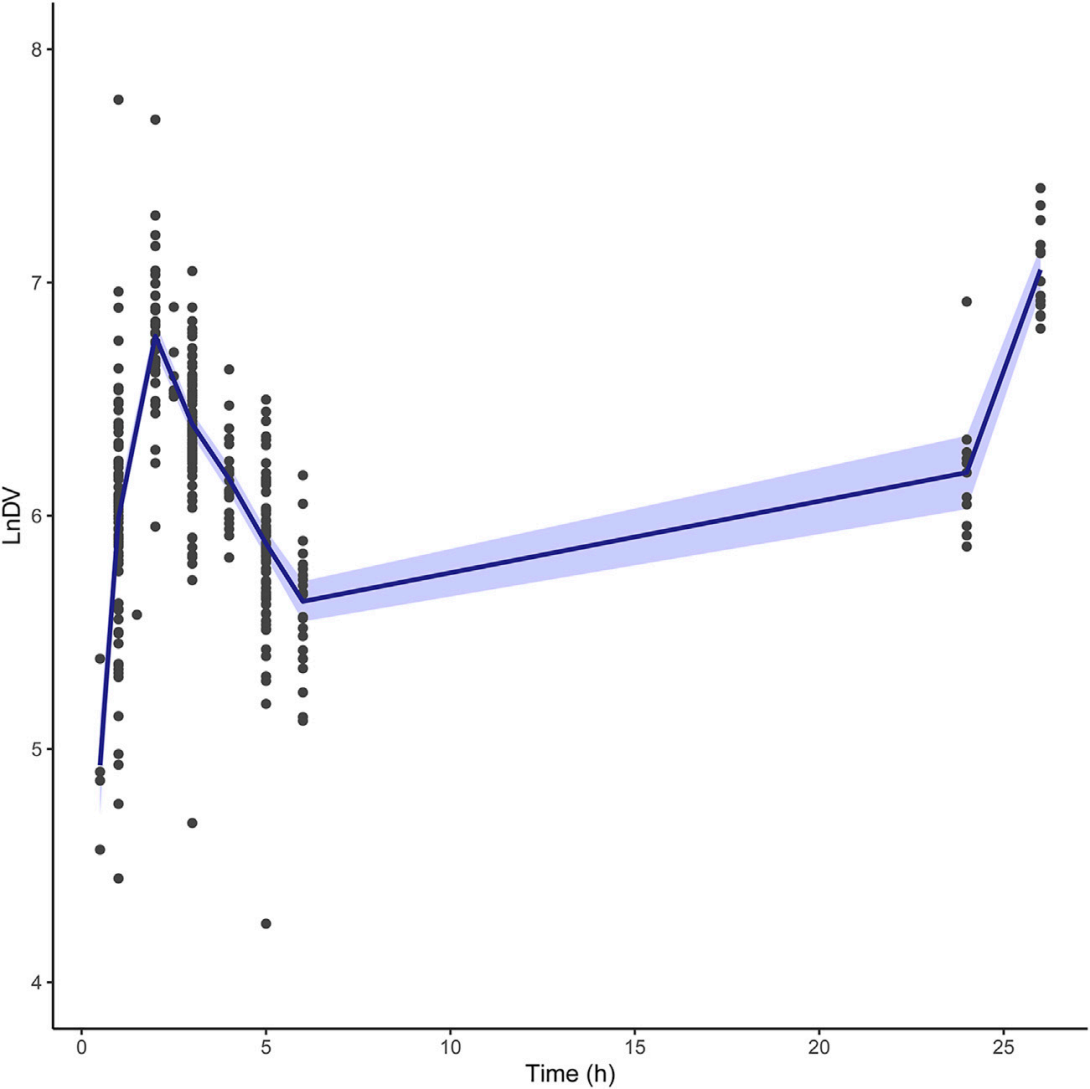
The LnDV vs. Time plot for external dataset. The middle solid blue line represents the mean LnDV. The semitransparent fields represent a 90% confidence interval for the mean.

In the seven published pop PK models, only the one built by Su et al. was two-compartment model. This may due to differences in PK sampling times. Most pop PK studies sampled at 0.5 h after the end of the infusion with the possibility of missing the fast distribution phase, while the sampling schedule in Su et al. was 0.25, 0.5, 1, 2, 2.25, 2.5, 3, 4, and 6 h after the start of the infusion for dose 1 or 9. If sufficient samples are collected in the fast distribution phase, the pop PK model may be developed as a two-compartment model. Another PK study with a dense sampling scheme also confirmed that BU fits a two-compartment model with a very rapid distribution phase (t_1/2α_ = 0.05 h), (Hassan et al., 1994). Given that the first sampling time in the external dataset was 0.5 h after the end of infusion, one-compartment model fitted better with our dataset. The LnDV vs. TIME plot was showed in Figure 6. The disposition for most individuals were observed a single slope.

Although the model built by Huang et al. was established based on a small population (20 subjects) and incorporated no covariates, it showed good predictability with our dataset of 110 patients. The reason may be found in the structural model of our dataset, in which the only incorporated covariate was BSA. However, the exponent for effect of BSA on CL was 1.05, much close to 1. In addition, the BSA range in Chinese patients is usually not wide. It can be considered that the impact of BSA on CL was not influential. Therefore, it could be accepted to guide individualized dosage adjustment of BU in Chinese patients because of the satisfied evaluation results.

It is important to explore the predictive ability of the published pop PK models in patients with extreme weight and specific GSTA_1_ genotypes, because it is usually these subjects with these extreme characteristics that need to adjust the dose. We examined the predictive performance of the models using obese patients (BMI ≥ 24, 31 subjects) and patients with GSTA_1_ *A/*B genotype (14 subjects). Similarly, the model built by Huang et al. showed better predictive performance than other models. For example, the MDPE was 6.21%, and the maximum F_20_ (64.57%) and F_30_ (77.35%) were also achieved in obese patients.

The Practice Guidelines Committee of ASBMT pointed that fludarabine, deferasirox, and metronidazole affected intravenous BU CL (Palmer et al., 2016). When BU was combined with oral or intravenous metronidazole, BU CL decreased by 46 and 57%, respectively (Gulbis et al., 2011; Chung et al., 2017). Fludarabine slightly affected the intravenous BU CL, with an average of 9.7% reduction (Yeh et al., 2012). However, others didn’t get the same results (Russell et al., 2002; de Lima et al., 2004). Co-administration with deferasirox led to a 1.5 times higher AUC (Sweiss et al., 2012). Phenytoin was usually used to prevent seizures when conditioning. It is reported that phenytoin had a higher CL of oral BU, however, the effect of phenytoin upon intravenous BU is limited (Kangarloo et al., 2012; Beumer et al., 2014). The effect of the conditioning regimen on BU CL was investigated by Huang et al. during the model development, but no significant change was observed (Huang et al., 2019).

The application of the Bayesian approach for dosage individualization has proven to be of value in clinical practice for several drugs (Brooks et al., 2020; Guo et al., 2020). It has the advantage of minimizing the need for monitoring of plasma drug concentrations, such as patient blood loss, pain and the cost of determining plasma drug concentration of multiple samples. For Bayesian forecasting, it is important to choose the most appropriate pop PK models and optimal sampling times for dosage prediction (Brooks et al., 2016). Based on the results of Bayesian forecasting, model built by Huang et al. (Huang et al., 2019) had better predictive performance. It can be considered as a qualified model to guide individualized dosing in our center. It seems that two prior concentrations are enough because more prior concentrations no longer improve predictive accuracy. The precision of prediction with four prior observations was decreased in our results, which might be due to lack of adequate patients with ≥ 5 observations.

Based on the model built by Huang et al. (2019), the dosage adjustment strategy for Chinese HSCT patients will execute as follows. Firstly, the initial dose will be calculated by typical CL times AUC_target_, which can be determined by physicians. Secondly, two blood samples will be collected randomly after the end of the first dose infusion and the measured concentrations will be used to get the individual CL (CL_ind_) through Bayesian forecasting using NONMEM software. Lastly, the dosage will be adjusted by multiplying CL_ind_ by AUC_target_.

The study has some limitations, including the lack of subjects with enough intensive samplings, as well as the fact that all the subjects came from the same center. A portion of the subjects had no genetic polymorphism information. Further studies are needed to increase the number of subjects and study centers, which would be helpful to get a more persuasive conclusion.

In conclusion, a total of seven published BU adult pop PK models were externally evaluated using an independent dataset from patients undergoing HSCT in our center. Based on prediction-based diagnostic and Bayesian forecasting, the model developed by Huang et al. (2019) showed accurate predictive performance. It can be built into computer programs to guide personalized dosing in our center. Further studies are needed to evaluate its performance in other centers in China. Bayesian forecasting indicated a potential application of quantified pop PK models to guide dosage adjustment. Based on the obvious differences between the adult model and the pediatric model, further external evaluation of pop PK models of BU in pediatrics is planned to be conducted.

## Data Availability

The raw data supporting the conclusion of this article will be made available by the authors, without undue reservation.
