# Supplementary material for: External Evaluation of Population Pharmacokinetic Models of Busulfan in Chinese Adult Hematopoietic Stem Cell Transplantation Recipients
Source: Front Pharmacol. 2022 Jul 7;13:835037. doi: 10.3389/fphar.2022.835037 (PMC9300831; doi:10.3389/fphar.2022.835037)
Supplement: Supplementary file 1 [file DataSheet1.docx]

## Table S1 Results of normalized prediction distribution errors (NPDE)

| **Models** | **Wilcoxon signed rank test*** | **Fisher test*** | **Shapiro-Wilks test*** | **Global test*** |
| --- | --- | --- | --- | --- |
| *Published Studies* |  |  |  |  |
| Choi et al. (2015) | 0.36 | 0.13 | 0 | 0 |
| Wang et al. (2015) | 0.02 | 0.54 | 0 | 0 |
| Choe et al. (2012) | 0.03 | 0.09 | 0 | 0 |
| Salinger et al. (2010) | 0.03 | 0.06 | 0.02 | 0.06 |
| Huang et al. (2019) | 0.03 | 0.46 | 0 | 0 |
| Sun et al. (2020) | 0.03 | 0.48 | 0 | 0 |
| Su et al. (2016) | 0.16 | 0.03 | 0 | 0 |
| *Impact of Model structures* |  |  |  |  |
| 1-CMT (Base Model) | 0.03 | 0.46 | 0 | 0 |
| 1-CMT+BSA | 0.01 | 0.45 | 0 | 0 |
| 2-CMT (Base Model) | 0.23 | 0.06 | 0 | 0 |
| 2-CMT+BSA | 0.26 | 0.03 | 0 | 0 |

*Data is expressed as P value.

1-CMT, one-compartment model; 2-CMT, two-compartment model; BSA, body surface area.

Articles after duplicates removed (n=425)

Articles obtained from PubMed (n=422)

Articles obtained from China National Knowledge Infrastructure and Wanfang Data (n=3)

Articles checked for eligibility (n=33)

Exclude not relevant for this study: Articles didn’t include a pop PK model of busulfan were excluded (n=392)

Articles excluded：

1. Pop PK models built in pediatrics (n=20)

2. Pop PK models built in both adults and pediatrics (n=4)

3. The incorporated factors in pop PK models were not applicable in our dataset (n=2)

Articles included in this study (n=7)

**Figure S1 Study flow diagram of the published pop PK studies review**


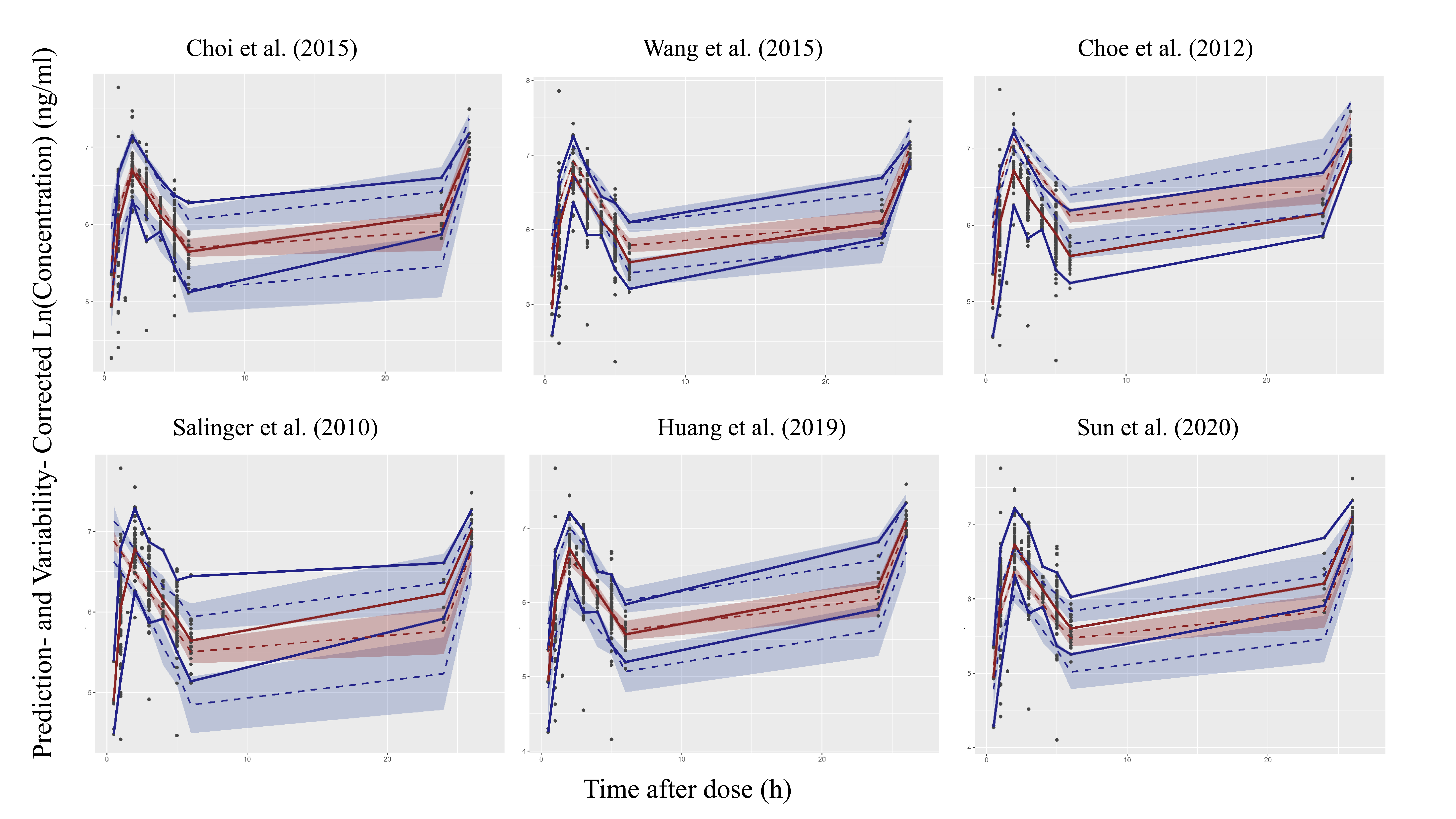


**Figure S2 Prediction- and variability- corrected visual predictive check (pvcVPC) plots in log scale of seven published population pharmacokinetic models (Salinger et al., 2010; Choe et al., 2012; Choi et al., 2015; Wang et al., 2015; Su et al., 2016; Huang et al., 2019; Sun et al., 2020). The middle dashed line represents the median prediction- and variability-corrected observations and predictions, respectively. The middle semitransparent field represents a simulation-based 95% confidence interval (CI) for the median. Upper and lower dash lines represent the corrected observed 95^th^ and 5^th^ percentiles and semitransparent fields represent a simulation-based 95% CI for the corresponding model predicted percentiles. The solid lines represent the median, 95^th^ and 5^th^ percentiles of observations. The model built by Su et al. cannot present VPC plot in log scale because the simulated values included non-positive values.**
